# Supplementary material for: Phylogenomics and antimicrobial resistance of the leprosy bacillus Mycobacterium leprae
Source: Nat Commun. 2018 Jan 24;9:352. doi: 10.1038/s41467-017-02576-z (PMC5783932; doi:10.1038/s41467-017-02576-z)
Supplement: Supplementary file 1 — Supplementary Information [file 41467_2017_2576_MOESM1_ESM.pdf]

Supplementary Note

Dating analysis

Dataset

Concatenated SNPs for each sample were used for tip dating analysis. We omitted strains with mutations in the *nth* gene because of their hypermutated genomes, which would interfere with the clock models in the Bayesian inference. Also, we removed highly mutated genes associated with drug resistant strains (**Table 1**) because they might contain mutations that arose from artificial selection during antibiotic treatments. Analysis was done in BEAST2 v2.4.4 [1]. Sites with missing data were included in the analysis because they contain valuable evolutionary signals and missing data are properly handled in BEAST2[2]. Constant (invariable) sites were also included as these contribute to better estimates of population size and branch lengths (https://groups.google.com/forum/#!topic/beast-users/-67kIqZEJf8). We included only unambiguous constant sites, i.e. loci where the reference base was called in all samples. These corresponded to A=230627, C=275179, G=279428, T=231694. Each BEAST run had between 50 M and 100 M MCMC steps to assure convergence and high ESS values for all indicators.

Substitution model

We used the BEAST package bModelTest v0.3.3[3] to infer the best substitution model that fits our data. Strict Clock and Constant Coalescent were used as clock and tree models. Best support resulted for the “123124” model after exploring all reversible models, or only the “transition/transversion split” models (**Supplementary Figure 4**). The “123124” model (A↔C = C↔G and A↔G = C↔T, **Supplementary Figure 5**) was used in subsequent BEAST analyses, although using the simpler HKY or the more complex GTR model produced similar results. bModelTest also supported invariable sites and gamma rate heterogeneity, so “hasGammaRates” and “hasInvariableSites” were used in all BEAST analyses.

Clock model

Mutation rates are not expected to vary significantly among closely related intraspecies taxa, so using the Strict Clock model is generally recommended for such cases[2]. To test whether a strict clock model should be rejected we performed an analysis in BEAST2 under the Log-Normal Relaxed Clock model and two population models, Constant Coalescent and Exponential Coalescent models. The mean coefficient of variation of the relaxed clock was 0.2035 (95% HPD 0.083-0.3256) under the Constant Coalescent model, and 0.1845 (95% HPD 0.0586-0.3086) under the Exponential Coalescent. In both cases, the marginal posterior distribution of the coefficient of variation of the relaxed clock extended down to zero, which means that the strict clock cannot be rejected for our data[2].

Root age under different models

To check for consistency of the dating analysis, we compared the estimates of the divergence time of TMRCA for all *M. leprae* strains under different clock and substitution models. Results remained consistent (**Supplementary Table 1**), indicating that the dataset has a strong clock signal and estimates are not affected by model priors.

***M. leprae* population size through time**

We used the Bayesian Skyline plot (BSP)[4] and the extended Bayesian skyline plot (EBSP)[5] in BEAST2 to infer changes in *M. leprae* population size through time. For the BSP, five and ten dimensions were used, which resulted in overlapping results. BSP resulted in a modest population size change of *M. leprae*, with no evidence of bottlenecks (**Supplementary Figure 6**). The first and last population size intervals in the Bayesian skyline analysis did not overlap, indicating population changes, but the separation was relatively small (4766 – 92708 vs. 94 – 4570) suggesting only a modest change of *M. leprae* population size through time. EBSP did not support a change of population size through time, which is not surprising for single locus data and relatively weak signals of population sizes in the data[2].

Supplementary Table 1 Estimated divergence times for the TMRCA for all *M. leprae* strains under different models in BEAST2.

Substitution model	Clock model	Population model	Mean root age (years ago)	95% HPD interval (years ago)
123124	Strict	Coalescent Constant	3697	2693 – 4878
123124	Strict	Coalescent Constant (with Discrete phylogeography)	3699	2731 – 4838
123124	Strict	Coalescent Exponential	3459	2568 – 4467
123124	Strict	Coalescent Bayesian Skyline (5 dimensions)	3544	2694 – 4497
123124	Strict	Coalescent Bayesian Skyline (10 dimensions)	3489	2692 – 4418
123124	Strict	Coalescent Bayesian Skyline Extended	3872	2814 – 5181
123124	Relaxed Log Normal	Coalescent Constant	3774	2492 – 5354
123124	Relaxed Log Normal	Coalescent Exponential	3395	2419 – 4525
bModelTest	Strict	Coalescent Constant	3702	2723 – 4844
HKY	Strict	Coalescent Constant	3692	2648 – 4797
HKY	Strict	Coalescent Exponential	3462	2553 – 4459
GTR	Strict	Coalescent Constant	3684	2685 – 4859
GTR	Strict	Coalescent Exponential	3445	2546 – 4436

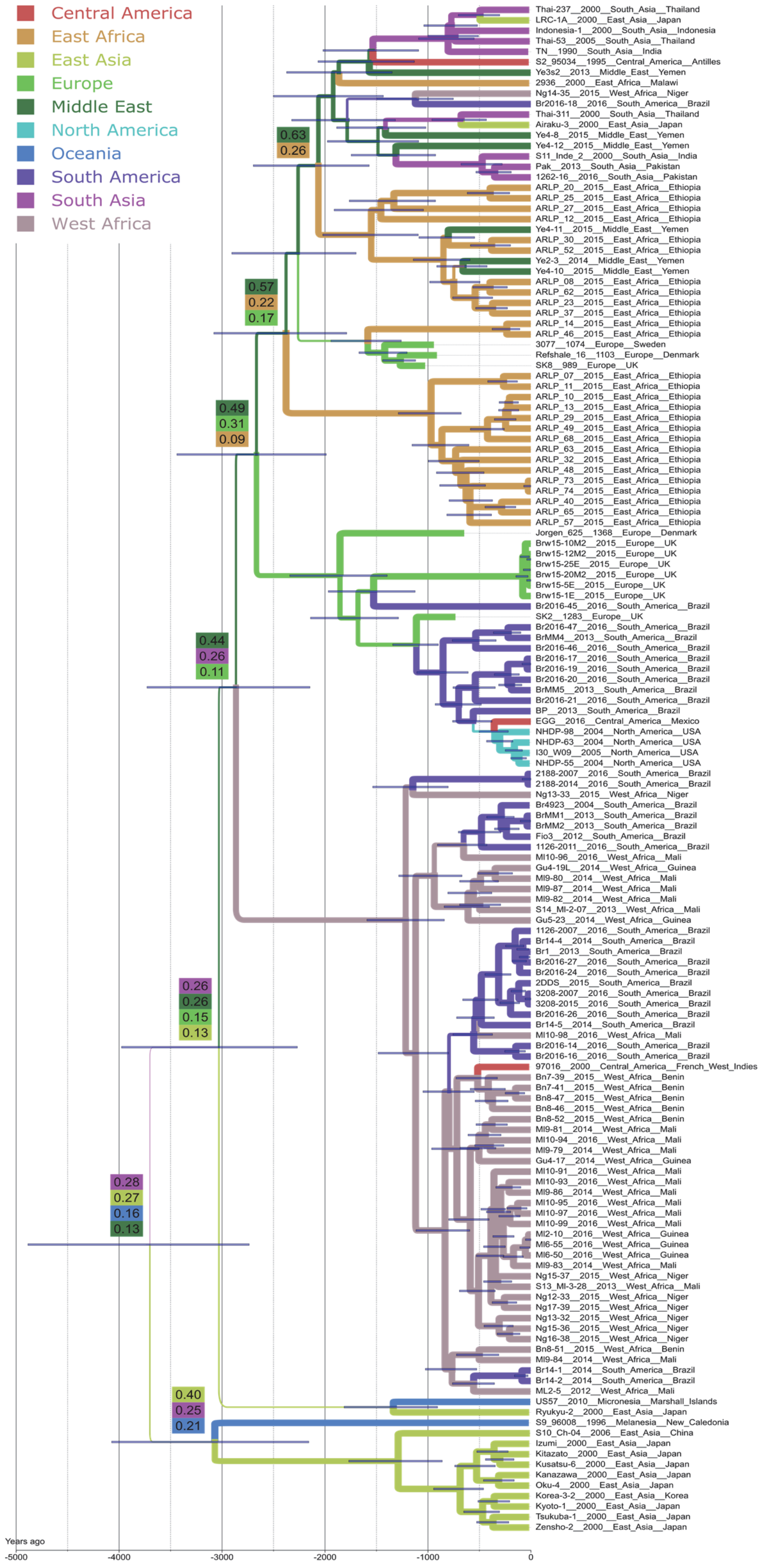

Supplementary Figure 1 Bayesian phylogenetic tree of 146 genomes of *M. leprae* calculated with BEAST 2.4.4. Hypermutated samples with mutations in the *nth* gene were excluded from the analysis. The tree is drawn to scale, with branch lengths representing years of age. Blue horizontal bars show the 95% Highest Posterior Density range of the age for each node. Samples were binned according to geographic origin as given in the legend. Location probabilities of nodes were inferred by the Discrete Phylogeny model and represented by line thickness, and values for the main basal nodes (note that the Discrete Phylogeny model can be influenced by sampling bias[2], therefore results should be interpreted with caution).

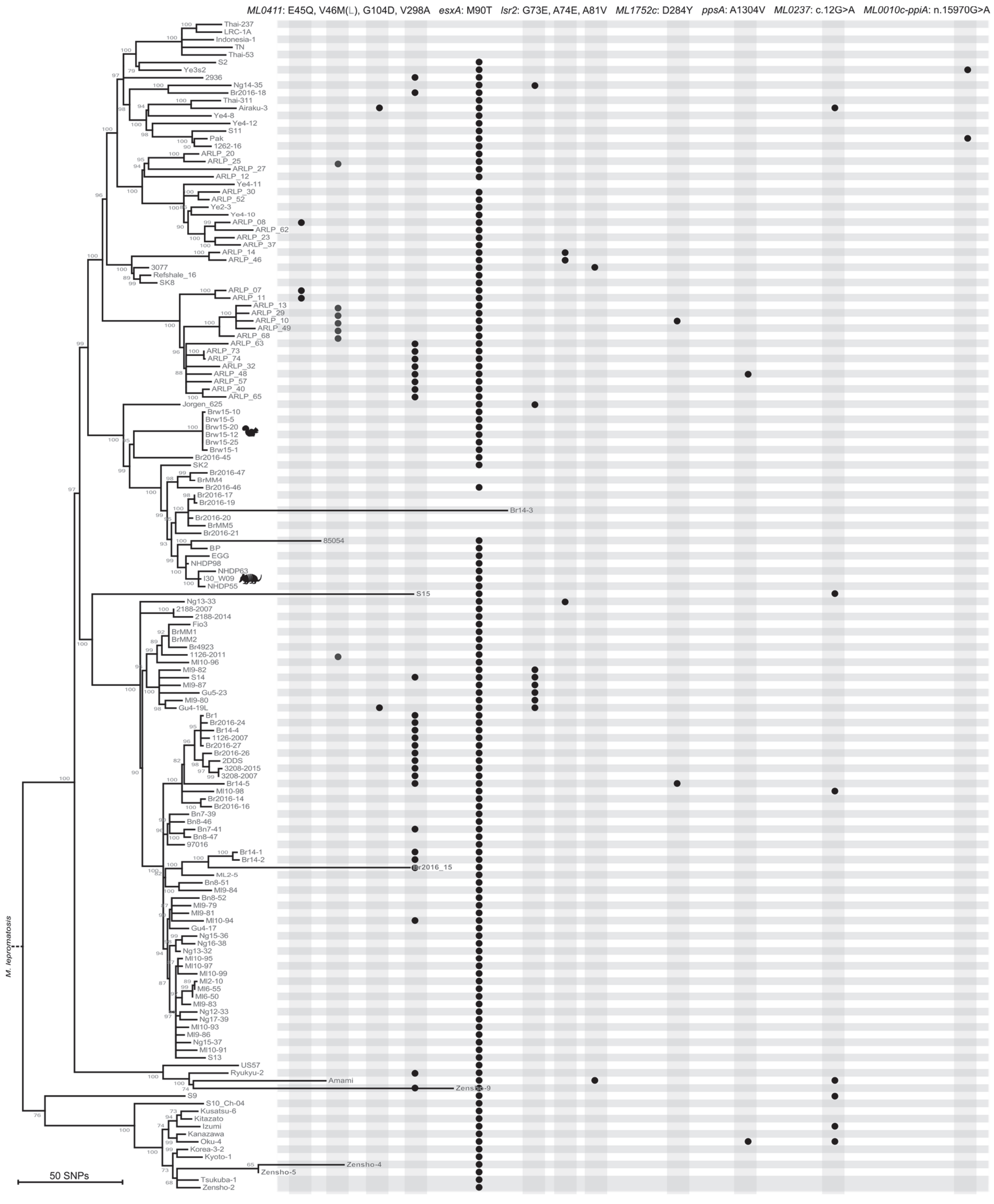

Supplementary Figure 2 Homoplasic sites detected in this study, excluding polymorphic VNTRs and homopolymeric tracts, and genes associated with drug resistance given in **Figure 4** in the main text.

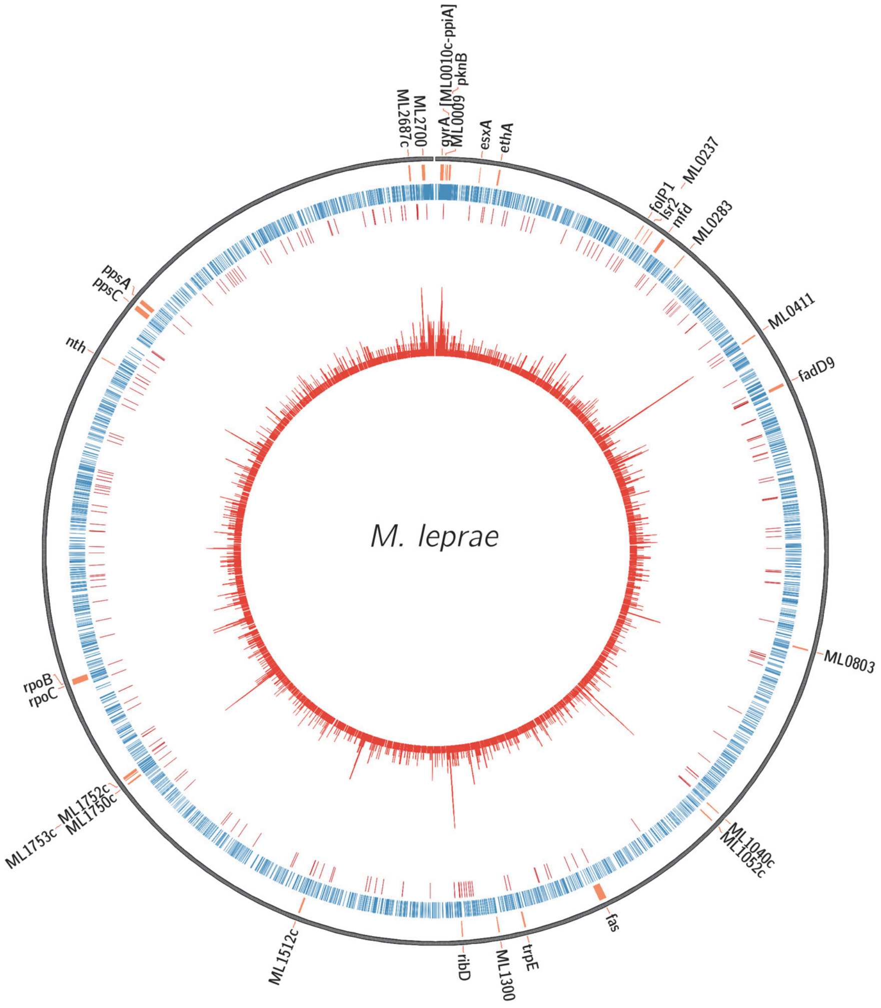

Supplementary Figure 3 Distribution of the mutations found in 154 strains for *M. lepreae*. Outer lane, highly polymorphic genes and genes with homoplasic mutations; 2nd lane (blue), SNPs; 3rd lane (red), InDels; inner lane, variant density per 50-base region.

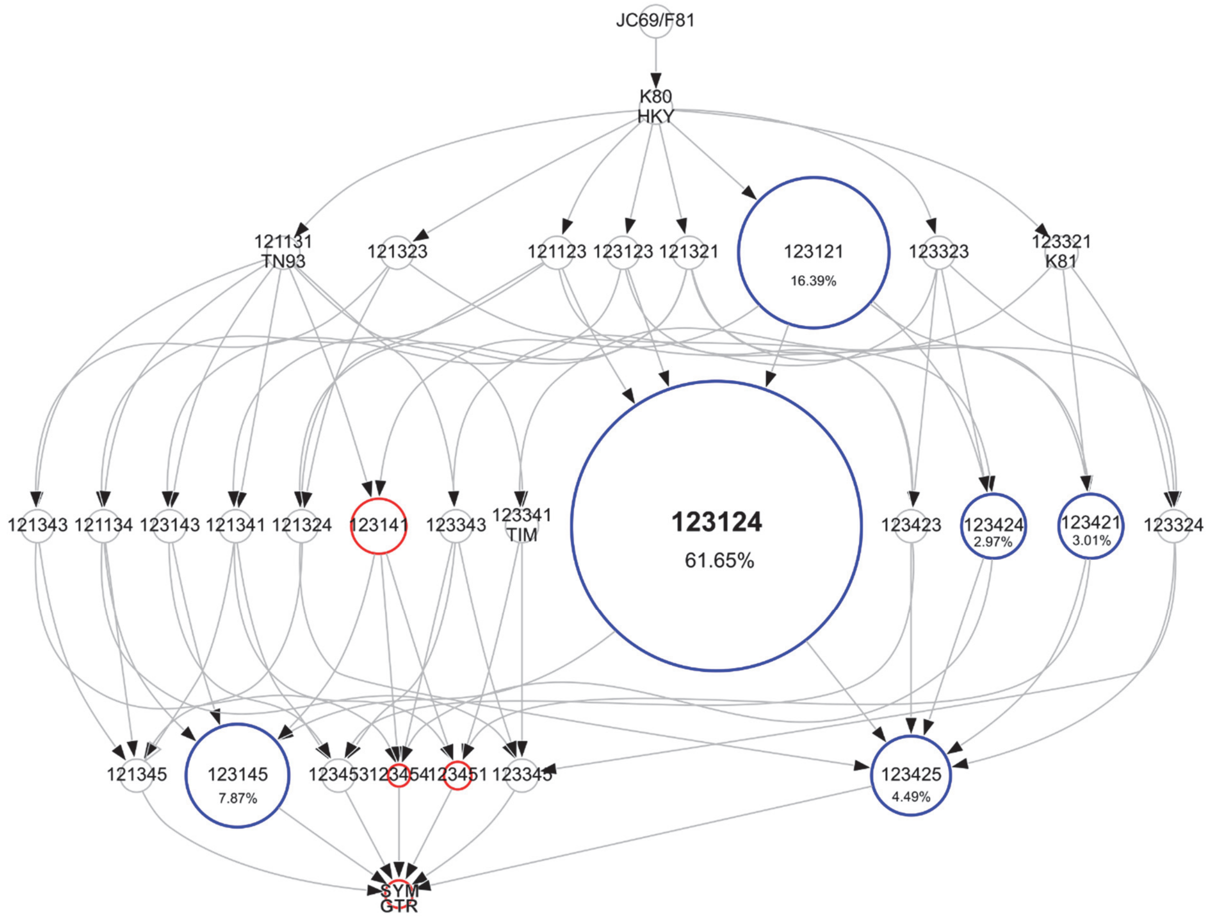

Supplementary Figure 4 Substitution model evaluation using the bModelTest tool in BEAST2. Models with blue circles are inside 95% HPD, red outside, and grey circles have 0.00% posterior support. Within each circle is the name of the substitution model, while blue circles also show the posterior support.

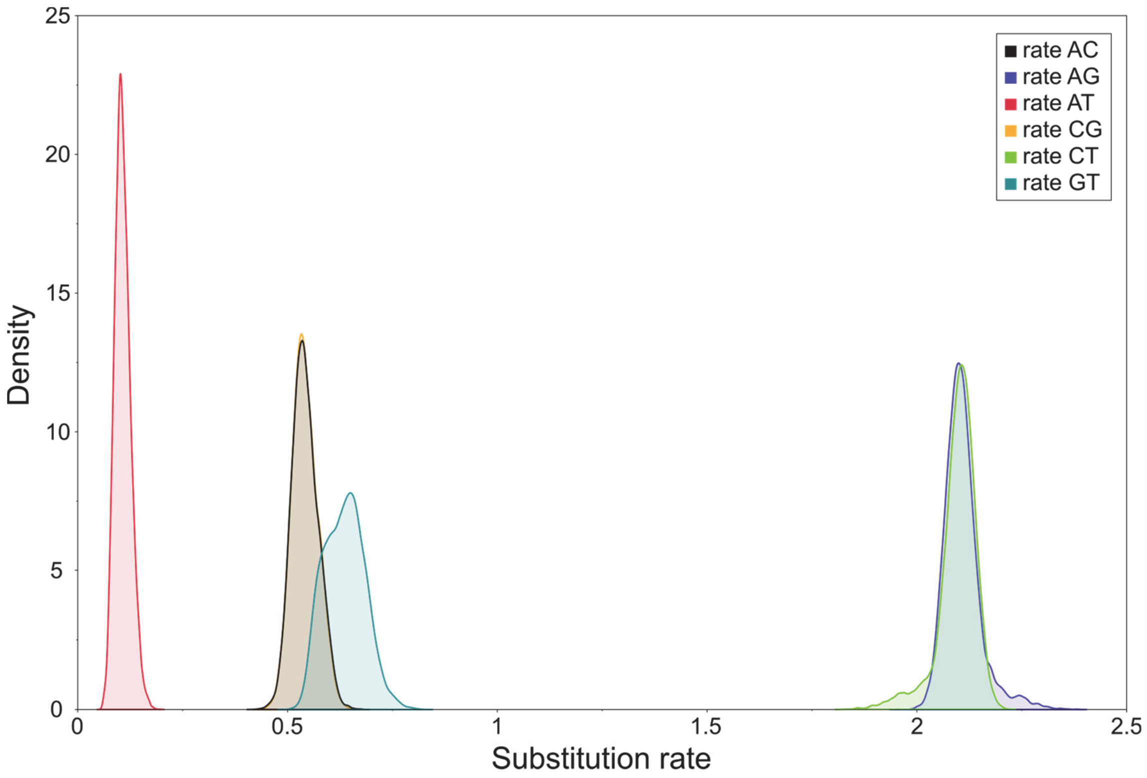

Supplementary Figure 5 Marginal posterior densities for the relative substitution rates in *M. leprae*, supporting the "123124" substitution model (A↔C = C↔G and A↔G = C↔T). Inferred in BEAST2 using the bModelTest tool.

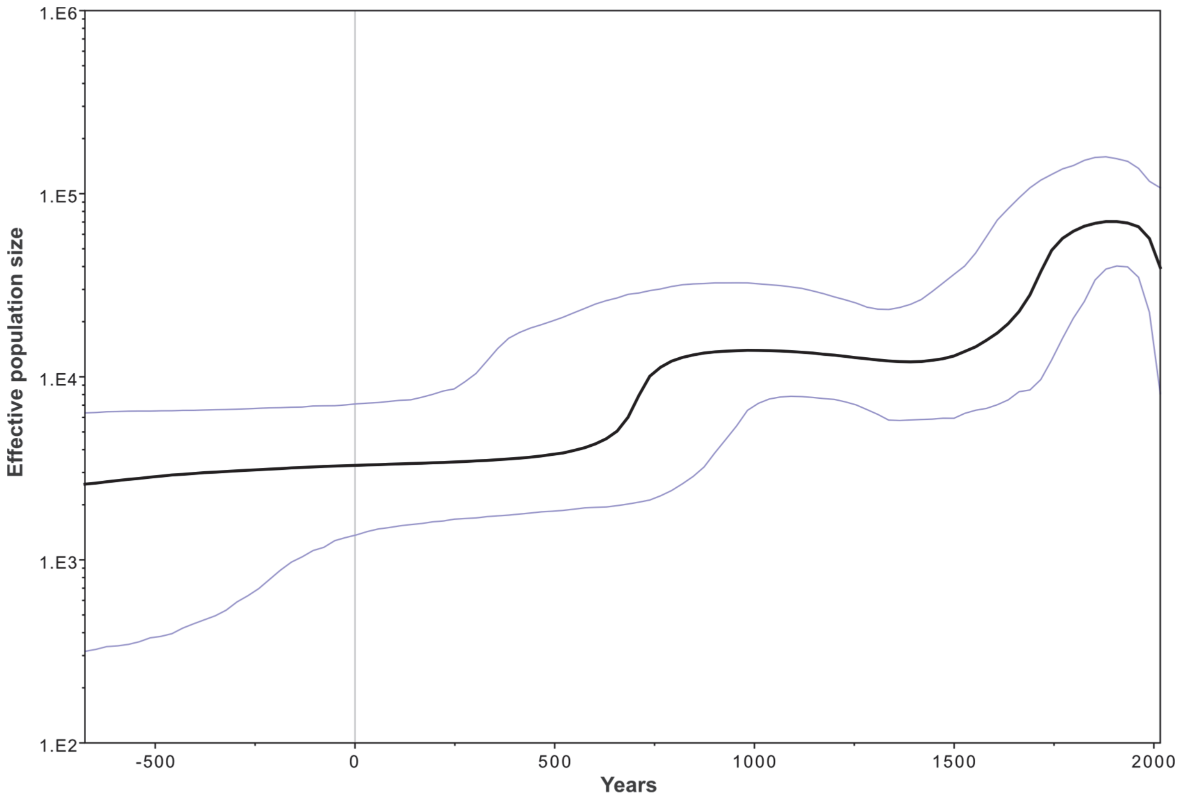

Supplementary Figure 6 Analysis of the effective population size of *M. leprae* using the Bayesian Skyline plot in BEAST2. Black line is the mean; blue lines indicate the 95% HPD boundaries.

Supplementary References

1. Bouckaert, R. *et al.* BEAST 2: a software platform for Bayesian evolutionary analysis. *PLoS Comput. Biol.* **10,** http://doi.org/10.1371/journal.pcbi.1003537 (2014).
2. Drummond, A. J. & Bouckaert, R. R. *Bayesian evolutionary analysis with BEAST*. (Cambridge University Press, 2015).
3. Bouckaert, R. R. & Drummond, A. J. bModelTest: Bayesian phylogenetic site model averaging and model comparison. *BMC Evol. Biol.* **17,** 42 (2017).
4. Drummond, A. J., Rambaut, A., Shapiro, B. & Pybus, O. G. Bayesian coalescent inference of past population dynamics from molecular sequences. *Mol. Biol. Evol.* **22,** 1185–1192 (2005).
5. Heled, J. & Drummond, A. J. Bayesian inference of population size history from multiple loci. *BMC Evol. Biol.* **8,** 289 (2008).
