# Supplementary material for: Phylogenomics and antimicrobial resistance of the leprosy bacillus Mycobacterium leprae
Source: Nat Commun. 2018 Jan 24;9:352. doi: 10.1038/s41467-017-02576-z (PMC5783932; doi:10.1038/s41467-017-02576-z)
Supplement: Supplementary file 3 — Description of Additional Supplementary Files [file 41467_2017_2576_MOESM3_ESM.pdf]

Description of Additional Supplementary Files

File Name: Supplementary Data 1
Description: Samples used in this study.

File Name: Supplementary Data 2
Description: SNPs and InDels identified in 154 M. leprae genomes.
